# Radiographic Findings in Patients with Medication-Related Osteonecrosis of the Jaw

**DOI:** 10.1155/2017/3190301

**Published:** 2017-03-02

**Authors:** Camila Lopes Cardoso, Carolina Arrabal Barros, Cláudia Curra, Luciana Maria Paes da Silva Ramos Fernandes, Solange de Oliveira Braga Franzolin, Joel Santiago Ferreira Júnior, Carlos César De Antoni, Marcos Martins Curi

**Affiliations:** ^1^Department of Oral Surgery, Universidade do Sagrado Coração, Bauru, SP, Brazil; ^2^Department of Oral Radiology, Universidade de São Paulo, São Paulo, SP, Brazil; ^3^Department of Biostatistics, Universidade do Sagrado Coração, Bauru, SP, Brazil; ^4^Department of Stomatology, Hospital Santa Catarina, São Paulo, SP, Brazil

## Abstract

A retrospective study was conducted of the records and panoramic radiographs of 35 patients treated with bisphosphonates (BP) and diagnosed with MRONJ. Panoramic radiography was used for evaluation, by two examiners, the following findings were subject of search: osteolysis (OT), cortical bone erosion (EC), bone sclerosis focal (FS) and diffuse (DS), bone sequestration (BS), thickening of lamina dura (TD), prominence of the inferior alveolar nerve canal (IAN), persisting alveolar sockets (SK), and the presence of a pathological fracture (PF). Medical information and staging were also recorded in order to correlate with radiographic findings. Bone sclerosis was the most frequent alteration, followed by OT and TD. The mandible was more affected than the maxilla. There was no significant difference between genders or significant correlation between the number of injuries with age and duration of BP usage. Considering the association between the radiographic findings and MRONJ staging, EC was predominant in stage 3 and DS in stage 2. IAN and PF demonstrated greater association with stage 3. In conclusion, the higher the clinical staging, the greater the severity of the bone alteration. Panoramic radiographic examination is a useful screening tool in patients submitted to antiresorptive therapy.

## 1. Introduction

Medication-related osteonecrosis of the jaw (MRONJ) is a well-known oral complication of antiresorptive or antiangiogenic agents in the oral and maxillofacial regions [1]. Staging of MRONJ is currently based on the classification proposed by the American Association of Oral and Maxillofacial Surgery (AAOMS), which is based on clinical and radiographic examinations [1]. An effective staging is of fundamental importance to the planning of treatment for MRONJ. However, the clinical manifestation of MRONJ sometimes presents with negligible superficial signs and does not always reflect the true extent of the underlying bone tissue. Clinical signs, such as bone exposure, infection, fistulae, mucosal erythema, and purulent drainage, do not always reflect the true extent of the disease in bone. Some studies have reported the most common radiographic findings of MRONJ using radiographic dental examinations, as follows: thickening of the lamina dura, diffuse osteosclerosis, osteolysis, presence of bone sequestrum, disruption of medullary trabecular bone, and alveoli with delayed remodeling [2–6]. Few MRONJ studies have been conducted with regard to the correlation between radiographic findings and staging of the disease [6]. Therefore, a retrospective study was conducted using records and panoramic radiographs of patients treated with bisphosphonates (BP) and diagnosed with MRONJ.

## 2. Material and Methods

A retrospective study was conducted using records and panoramic radiographs from the databases of the Hospital Santa Catarina and University of Sagrado Coração, São Paulo, Brazil, encompassing a period of 12 years (2003–2015). The inclusion criterion was the patient presenting MRONJ associated with bisphosphonate therapy. Diagnosis and staging were based on the publication by the AAOMS of its 2014 guidelines [1]. Patients undergoing head and neck radiotherapy were excluded from this study. The data was collected from the patient's medical records as follows: age, gender, type of systemic disease, type of BP, duration of BP treatment, site of the MRONJ, drug administration protocol, and MRONJ staging. The aim of this study was to identify the radiographic findings of MRONJ and correlate them with the AAOMS clinical staging system. Stage 0 included patients with clinical signs of osteonecrosis other than exposed bone. Stage 1 included patients with exposed necrotic bone but no signs of infection. Stage 2 included patients with exposure of necrotic bone together with signs and symptoms of infection, and stage 3 included patients with exposed necrotic bone and an extraoral fistula, sequestration, or mandibular fracture. Radiographic features consisted of the use of panoramic radiography.

Radiographic evaluation was performed by two calibrated examiners (1 and 2). For the evaluation, the arches were divided into sextants (1, 2, 3: maxilla and 4, 5, 6: mandible) based on a previously described methodology [5]. Osteolysis (OT), cortical bone erosion (EC), bone sclerosis focal (FS) and diffuse sclerosis (DS), bone sequestration (BS), thickening of the lamina dura (TD), prominence of the inferior alveolar nerve canal (IAN), persisting alveolar sockets (SK), and the presence of pathological fracture (PF) were investigated. MRONJ staging and the patient's medical history were also recorded for correlation with the radiographic findings. Data from the measurements were organized in Excel tables (Microsoft Office Excel, Redmond, WA, USA) and submitted to SigmaPlot software (SigmaPlot, San Jose, CA, USA) version 12.3. The agreement between the different factors evaluated by the examiners (1 and 2) was interpreted by a Kappa inter-rater test. For the association between nominal variables we used the statistical test Chi-square and Fisher's exact statistical tests. Pearson correlation coefficients (nominal variables) and Spearman correlation (ordinal variable) were used for correlations. The agreement between the AAOMS staging system and the radiographic findings for the detection of bone disease was evaluated by calculating the proportion of patients in each AAOMS stage. Data were analyzed regarding normal distribution (Shapiro-Wilk test and equal variance assumption) and subsequentl the one-criterion analysis of variance test (Score Factor) was adopted with the radiographic findings.

## 3. Results

Thirty-five patients were selected for this study, in accordance with the inclusion criteria. Demographic data are shown in Table 1. Of the 35 patients, 8 (22.9%) were male and 27 (77.1%) female. The mean age was 59.5 years (ranging from 24 to 95 years), with a standard deviation of 13.7 years. Twenty-eight cases (72%) occurred in the mandible, and seven cases (28%) occurred in the maxilla. All patients had a diagnosis of cancer, except for two women who had osteoporosis. Patients were under BP treatment with zoledronic acid (27 patients/77.1%), pamidronate (3 patients/8.57%), association of zoledronic acid and pamidronate (2 patients/5.71%), alendronate (2 patients/5.71%), and ibandronate (1 patient/2.85%). The median total duration of BP therapy was 42 months (ranging from 6 to 288 months, standard deviation of 50 months). BP was administered intravenously to 32 (91.4%) patients and the other three patients (8.6%) received it orally. Regarding the clinical staging of MRONJ, 4 (11,4%) patients were stage “0,” 8 (22,9%) patients were stage “1,” 19 (54,3%) were stage “2,” and 4 (11,4%) were stage “3.”

There was total agreement among the examiners (Kappa = 1). The Chi-square statistical test showed no statistically significant difference between genders (*p* = 0.24). With regard to the sextant most affected, it was observed more frequently in 4, 5, and 6 (mandible) with a statistically significant difference (*p* < 0,00001) The Chi-square test recorded *p* < 0.00001. However, with gender, despite differences in absolute values, the statistical test showed no difference (*p* = 0.39).

The most significant radiographic finding was the bone sclerosis in all patients (19 focal sclerosis and 16 diffuse sclerosis) and osteolysis in 28 patients (Table 2). Other radiographic findings such as thickening of lamina dura (17 patients) and prominence of the inferior alveolar nerve canal (15 patients) were frequently found in the MRONJ region.

There was no correlation between patient's age and the number of lesions (Pearson correlation = 0.012207, *p* = 0.94451), the length of drug use, and the number of radiographic findings (Pearson correlation = 0.17975; *p* = 0.30148). Considering the association between the radiographic findings and MRONJ staging, there was a significant difference in the following findings: cortical erosion (*p* < 0.008), predominantly in stage 3 (*p* = 0.77); diffuse sclerosis (*p* = 0.003), predominantly in stage 2; prevalence of IAN (*p* < 0.001) and pathologic fracture *p* ≤ 0.001, with a greater association with stage 3 (*p* = 0.875). The radiographic findings and the association with the staging of MRONJ are shown in Table 3.

## 4. Discussion

MRONJ is a significant oral complication, only recently described in the literature. It was first reported in 2003 [7]. Considering the staging system for MRONJ, some authors have recommended a new staging based on clinical manifestations and imaging findings. A wide spectrum of radiographic features has been reported in MRONJ. These findings include bone sclerosis, thickening of lamina dura, erosion of the adjacent cortical bone, periosteal reaction, disruption of spinal trabecular bone, tooth alveolus with delayed remodeling, prominence of the IAN canal, sequestration, and pathological fracture [2–5]. In the present study, 35 patients with a diagnosis of MRONJ, most of whom were treated with zoledronate, were radiographically evaluated for bone abnormalities. A wide spectrum of radiographic findings was observed in all patients, regardless of the clinical stage of the disease. In agreement with previous studies, the mandible was the anatomical region that most frequently revealed radiographic abnormalities probably because it is the region most affected by MRONJ.

The most prevalent radiographic findings were bone sclerosis and osteolysis, similar to other studies [6, 8]. Authors reported a positive relationship between the increased degree of bone sclerosis and the clinical severity of the disease [9]. In advanced disease, radiographic evidence of mandibular canal narrowing was also reported [9]. Considering the association between the radiographic findings and MRONJ staging, in this study, a greater degree of severity of bone alteration was observed in stage 3, which is the most advanced. 75% of these patients demonstrated pathological fracture. Also, patients, presenting with stage 2, revealed significant bone alterations such as prominence of the inferior alveolar nerve canal.

The radiographic findings often do not show any signs on clinical examination, or symptoms, which justify additional radiographic assessment. Most of the studies into complementary imaging exams for MRONJ discussed the accuracy of the conventional radiographs. However, the panoramic radiograph is the most requested imaging examination, so the knowledge of the main radiographic findings therein is extremely valuable to the clinician. This fact highlights the importance of the present study [4, 5, 10].

The panoramic radiography is a very common examination for patients with MRONJ and has been routinely used to establish the diagnosis and also to plan treatment. It is a simple examination and an easily accessible tool for clinicians and oral surgeons for the initial evaluation and diagnosis of MRONJ. It permits a quick viewing of the areas of concern and serves as the initial radiological screening of patients who present for evaluation of MRONJ. Moreover, the panoramic radiograph is less expensive and presents lower radiation exposure than compared to computed tomography, which has demonstrated greater sensitivity in the diagnosis of MRONJ when compared with the panoramic radiograph. The radiographic findings, such as thickening of lamina dura, osteolysis, bone sclerosis, and poor alveolar healing, may be capable of detection in these examinations.

Some imaging findings of MRONJ may be very similar to other bone diseases such as osteomyelitis, osteoradionecrosis, and bone metastases, so the medical history of the present illness and physical examination are essential to the determination of the final diagnosis and treatment plan.

With more than a decade of knowledge of this clinical entity, there are several reports in the literature about MRONJ with stage 0 disease (clinical symptoms without exposed necrotic bone) considered at risk for MRONJ [10–13]. Case series reports, describing radiographic findings in patients with symptomatic stage 0, have contributed to efforts to characterize radiographic patterns that may precede development of MRONJ in at-risk populations [10–14]. The radiographic findings observed in MRONJ stages 1 to 3 have also been similarly described for stage 0 disease. Some authors have postulated that thickening of lamina dura, lack of bone repair/persisting of alveolar socket, and focal osteosclerosis may be early radiographic features of preclinical MRONJ. Recognition of early radiographic changes may help identify patients potentially at risk for MRONJ. In this context, the knowledge of the imaging findings of MRONJ may be essential for a correct diagnosis, the establishment of an appropriate treatment plan, and consequently a better prognosis.

This assessment does not have a large control group of patients using bisphosphonates, and without a diagnosis of MRONJ, so it is not possible to address causality. So we cannot, therefore, exclude the possibility that the radiographic findings are related to previous dental problems or anatomical variations before bisphosphonate therapy was begun. However, the radiographic findings identified in this study are supported by previously published works on patients with a diagnosis of MRONJ. Better knowledge of radiographic findings can help in the planning and handling of patients with a diagnosis or who are at risk of MRONJ.

## 5. Conclusions

In conclusion, the higher the clinical staging, the greater the severity of bone alteration. Panoramic radiography examination is a useful screening tool that may improve early diagnosis of MRONJ, establishing a preventive approach for this complication.

## Figures and Tables

**Table 1:** Data of 35 patients with MRONJ.

Patients	Age	Gender	Systemic disease	Bisphosphonate	Treatment (months)	Drug administration	Stage
1	45	Male	Cancer	Aredia	12	Intravenous	3
2	45	Male	Cancer	Zometa	20	Intravenous	0
3	54	Female	Cancer	Aredia/Zometa	84	Intravenous	0
4	42	Male	Cancer	Aredia	14	Intravenous	3
5	54	Female	Cancer	Zometa	28	Intravenous	1
6	52	Female	Cancer	Zometa	8	Intravenous	2
7	54	Female	Cancer	Zometa	108	Intravenous	2
8	68	Female	Osteoporosis	Fosamax	80	Oral	2
9	52	Female	Cancer	Zometa	48	Intravenous	1
10	49	Female	Cancer	Zometa	18	Intravenous	2
11	59	Female	Cancer	Zometa	8	Intravenous	1
12	39	Female	Cancer	Zometa	6	Intravenous	2
13	59	Female	Cancer	Aredia/Zometa	84	Intravenous	2
14	79	Female	Osteoporosis	Fosamax	56	Oral	2
15	52	Female	Cancer	Aredia	26	Intravenous	0
16	64	Female	Cancer	Zometa	30	Intravenous	2
17	74	Female	Cancer	Zometa	48	Intravenous	2
18	90	Female	Cancer	Zometa	12	Intravenous	2
19	57	Female	Cancer	Zometa	36	Intravenous	2
20	66	Female	Cancer	Zometa	36	Intravenous	2
21	64	Female	Cancer	Zometa	72	Intravenous	1
22	24	Male	Cancer	Zometa	18	Intravenous	1
23	77	Male	Cancer	Zometa	12	Intravenous	2
24	52	Male	Cancer	Zometa	22	Intravenous	1
25	65	Female	Cancer	Zometa	24	Intravenous	3
26	95	Female	Cancer	Bonviva	36	Oral	2
27	67	Female	Cancer	Zometa	288	Intravenous	2
28	58	Female	Cancer	Zometa	30	Intravenous	2
29	64	Female	Cancer	Zometa	6	Intravenous	0
30	50	Male	Cancer	Zometa	24	Intravenous	3
31	56	Female	Cancer	Zometa	24	Intravenous	1
32	64	Female	Cancer	Zometa	30	Intravenous	2
33	65	Female	Cancer	Zometa	48	Intravenous	2
34	60	Female	Cancer	Zometa	48	Intravenous	2
35	70	Male	Cancer	Zometa	16	Intravenous	1

**Table 2:** Radiographic findings of 35 patients with MRONJ. Osteolysis (OT), erosion of cortical bone (EC), bone sclerosis focal (FS) and diffuse sclerosis (DS), bone sequestration (BS), thickening in the lamina dura (TD), prominence of the inferior alveolar nerve canal (IAN), persisting alveolar sockets (SK), and presence of pathological fracture (PF).

Patients	Stage	OT	EC	FS	DS	SQ	TD	IAN	SK
1	3	+	+	−	−	−	+	−	+
2	0	+	−	−	−	−	+	−	−
3	0	+	−	+	−	−	+	−	+
4	3	+	+	+	+	−	−	+	−
5	1	+	−	−	−	−	−	−	+
6	2	+	−	−	+	+	−	+	−
7	2	+	−	+	+	−	+	+	−
8	2	+	+	+	+	+	−	+	+
9	1	+	−	+	−	+	−	−	−
10	2	−	−	+	−	−	+	−	−
11	1	−	−	+	−	−	+	−	−
12	2	−	−	+	+	−	+	+	−
13	2	+	−	+	−	−	+	−	−
14	2	+	+	−	+	+	−	+	+
15	0	+	−	−	−	+	−	−	−
16	2	−	+	+	+	−	+	+	+
17	2	+	−	+	+	−	−	+	−
18	2	+	−	−	+	−	−	+	−
19	2	+	−	+	+	−	+	+	−
20	2	+	+	+	+	−	+	+	−
21	1	+	−	−	−	−	+	−	−
22	1	−	−	+	−	−	−	−	+
23	2	+	−	+	−	−	−	−	−
24	1	+	−	−	−	−	−	−	+
25	3	+	+	−	−	+	−	−	−
26	2	+	+	−	+	−	−	+	−
27	2	−	−	+	+	−	+	+	−
28	2	+	+	+	−	−	+	−	−
29	0	+	+	−	+	−	−	−	−
30	3	+	+	+	−	−	−	−	+
31	1	−	−	−	−	−	+	−	−
32	2	+	−	−	+	+	+	+	+
33	2	+	−	−	+	+	−	+	+
34	2	+	+	+	−	−	+	−	−
35	1	+	−	−	−	+	−	−	+
*Total (n*)		*28*	*12*	*19*	*16*	*9*	*17*	*15*	*12*

**Table 3:** Number and percentage of radiographic findings in each stage.

Stage	OT (%)	EC (%)	FS (%)	DS (%)	SQ (%)	TD (%)	IAN (%)	SK (%)	PF (%)
0	4 (100)	1 (25)	1 (25)	1 (25)	1 (25)	2 (50)	0 (0)	1 (25)	0 (0)
1	5 (62,5)	0 (0)	3 (37,5)	0 (0)	2 (25)	3 (37,5)	0 (0)	4 (50)	0 (0)
2	15 (78,9)	7 (36,8)	13 (68,4)	**14 (73,6)** ^**∗**^	5 (26,3)	11 (57,9)	**14 (73,7)** ^**∗**^	5 (26,3)	0 (0)
3	4 (100)	**4 (100)** ^**∗**^	2 (50)	1 (25)	1 (25)	1 (25)	1 (25)	2 (50)	**3 (75)** ^**∗**^

^**∗**^Radiographic findings that presented a significant association with the clinical stage.
